# Medication administration error and contributing factors among pediatric inpatient in public hospitals of Tigray, northern Ethiopia

**DOI:** 10.1186/s12887-018-1294-5

**Published:** 2018-10-10

**Authors:** Zeray Baraki, Mebrahtu Abay, Lidiya Tsegay, Hadgu Gerensea, Awoke Kebede, Hafte Teklay

**Affiliations:** 1 Department of Neonatal Nursing, School of Nursing, College of Health Sciences, Aksum University, Aksum, Ethiopia; 2 Department of Epidemiology and Biostatistics, School of Public Health, College of Health Sciences, Aksum University, P. O. Box: 298, Aksum, Ethiopia; 3 Department of Biomedical Sciences, School of Medicine, College of Health Sciences, Aksum University, Aksum, Ethiopia

**Keywords:** Medication administration error, Pediatrics, Inpatient, Tigray, Ethiopia

## Abstract

**Background:**

Medication administration error is a medication error that occurs while administering a medication to a patient. A variety of factors make pediatrics more susceptible to medication errors and its consequences. In low-income countries, like Ethiopia, there is no sufficient evidence regarding medication administration error among pediatrics. The aim of this study is, therefore, to determine the magnitude and factors associated with medication administration error among pediatric population.

**Methods:**

A prospective observational based cross sectional study design was conducted from January to April 2017. Data collection was done using pre-tested structured questionnaire and blind observation checklist to health professionals in charge of administering selected medications. A total of 1282 medication administrations were obtained using single population proportion formula from patients in the selected public hospitals and the samples were selected using multistage sampling technique. Multivariable logistic regression using odds ratio and 95% confidence interval was used to determine the relationship between the independent and dependent variables. Variables with *p*-value < 0.05 were considered as independent factors for medication administration error.

**Result:**

A total of 1251 medication administrations were observed from 1251 patients. The occurrence of medication administration error was 62.7% with 95% CI (59.6%, 65.0%), wrong dose being the most common type of medication administration error with an occurrence rate of 53.7%. Medications administered for pediatric patients less than 1 month age, administered by bachelor degree holder health professionals, prepared in facilities without medication preparation room, prepared in facilities without medication administration guide and administer for patients who have two or more prescribed medications were more likely to have medication administration error than their counterparts with AOR (95% CI) of 7.54(2.20–25.86), 1.52 (1.07–2.17), 13.45 (8.59–21.06), 4.11 (2.89–5.85), and 2.42 (1.62–3.61), respectively.

**Conclusion:**

This study has revealed that there is high occurrence of medication administration error among pediatric inpatients in public hospitals of Tigray, Northern Ethiopia.. Age of patients, educational level of medication administrators, availability of the medication preparation room and guide, and the number of medications given per single patient were statistically significant factors associated with occurrence of medication administration error.

**Electronic supplementary material:**

The online version of this article (10.1186/s12887-018-1294-5) contains supplementary material, which is available to authorized users.

## Background

Medication administration has been defined by the Nursing Interventions Classification (NIC) as “preparing, giving and evaluating effectiveness of prescribed and non-prescribed medications; whereas a medication administration error is a medication error that occurs while administering a medication to a patient” [1]. The National Coordinating Council for Medication Error Reporting and Prevention (NCC MERP) states that “A medication error is any preventable event that may cause or lead to inappropriate medication use or patient harm while the medication is in the control of the health care professional, patient, or consumer” [2]. Despite the existence of increased levels of awareness and developments in technology designed to reduce such errors, high rate of medication error continue over the past decade [3].

The major consequences of medication administration errors (MAE) are patient morbidity and mortality. It can, indirectly, also affect patients, families and health care providers by cost implications, prolonged hospital stays and psychological impact since errors erode public confidence to health care services**.** Medication administration errors are potentially more harmful and have a higher incidence rate in the pediatric population than in the adult population. The rate of MAE with potential for injury within pediatric health care was 1.1%, which is three times higher than in a separate corresponding hospital study on adults, which revealed only 0.35% [4]. One of the factors that make the pediatric population more susceptible to medication errors include availability of different dosage forms of the same medication, which can lead to dosing errors. Unlike adults, most medication dosing of pediatric patients are based upon body weight, which requires a dosage calculation and hence can expose to an error [5]. Furthermore, children, in comparison to adults, are often unable to adequately communicate when they are experiencing an adverse effect and have a limited internal physiological capacity to buffer medication errors [5].

The prevalence of MAE is still high even in developed countries like United States of America (USA). Its prevalence among hospitalized pediatric patients in USA was 67% in 2004; 42,000 pediatric inpatients experience a preventable administration error, 21% of which are caused by MAE [6, 7]. In the UK, in 2012, among acutely admitted patients to hospital 178 of 6821 children had an adverse drug reaction because of MAE [8]. Similarly, in the Latin American country Argentina, for a total of 1174 observed medication administrations in neonatal and pediatric intensive care units wards, 99 had MAE [9]. In India, 313(68.5%) out of 457 medications administered had MAE [10]. In Nigeria, between July 2006 and December 2007, there have been 40 suspected adverse drug reactions (ADRs) out of 53 administered medications [11]. In Ethiopia, in 1020, from a total of 52 patients who had a total of 218 medication administrations, 196(89.9%) MAEs were occurred [12].

Although MAEs of all sorts are investigated throughout the developed world, the issue has only lightly been explored in the low-income countries like Ethiopia. There is dearth of in-depth information regarding a problem and contributing factors of MAE, particularly in a hospitalized pediatric population of Ethiopian health institutions. Hence, this study was intended to assess occurrence of MAE and associated factors among pediatric patients who admitted in selected public hospitals of Tigray, Ethiopia.

## Methods

### Study design and setting

In Central, Northwest and West zones of Tigray region, Ethiopia, there are 19 hospitals, six of which are public general hospitals. A prospective observation-based cross sectional study was carried out in these six public general hospitals of from September 2016 to August 2017.. Each of the public general hospitals serves for about 1–1.5 million population [13].

### Study population and sampling

The source populations are all hospitalized pediatric patients who were admitted in the pediatric ward, pediatric ICU and neonatal ICU of public general hospitals found in the selected Zones of Tigray region, northern Ethiopia. The study populations are all sampled hospitalized pediatric patients who were admitted in the pediatric ward, pediatric ICU and neonatal ICU of the public general hospitals.

The sample size was determined based on a single population proportion (p) formula n = [(z∞/2)2 p(1-p)]/ d^2^, with the assumptions of 95% of confidence level, 5%α, 2% margin of error and 89.9% occurrence of MAE, from a study conducted in Jima University specialized hospital, Jima, Ethiopia [12]. By using a design effect of 1.4 and 5% non-response rate, a total sample size of 1282 medication administrations was obtained. Using the multistage sampling technique, out of the seven zones in the region, three zones were selected by the simple random sampling method and then six hospitals were proportionally selected from the three zones. Allocation of the sample among the six hospitals was done proportional to the number of expected admissions of pediatric patients in each hospital. Finally, sampling frames of medication administration were then prepared from the pediatric and neonatal units of each hospitals and simple random sampling method was used to draw one sample administered medication for each patient.

### Data collection tool and quality assurance mechanisms

Data were collected through an observational checklist from the health professionals in charge of administering medications to observe the procedure of administration and interviewee- administered-structured-questionnaire was used to assess the socio-demographic and experience related factors of health professionals as well as socio-demographic factors of patients. The tool contains four components, part I (socio-demographic variables), part II (medication related variables), part III (facility and equipment related variables) and part IV (medication administration related variables. The questionnaire contains open and closed ended questions, which was adopted contextually from the WHO standard (right) of medication administration and NCC MERP recommendation for safe medication administration [14–16]. The data was collected by six midwives and twelve nurses, who were following their MSc during the data collection period, under supervision of six MSc holder nurses,. The data collection period ranged from January 10, 2017 to April 10, 2017 (Additional file 1).

To assure data quality, training was given for the data collectors by the principal investigator for three consecutive days. The data collection tool was pre-tested on 100 medication administrations and all corrections and amendment were considered 2 weeks prior to the actual data collection period in three primary hospitals.. Health professionals who were going to be observed while administering medications to each patient were informed about the work prior to the commencement of data collection, but the entire purpose of the study was not disclosed in order to ensure that the findings are unbiased. Six supervisors, on a daily bases, reviewed and checked the collected data for completeness, clearness and consistency and if there were any incorrectly filled and missed data. In cases of such findings they were sending back for immediate correction.

### Variables in the study

The outcome variable, medication administration status, is a binary outcome categorized as with MAE and without MAE. The independent variables include: patient related factors (age, sex, weight, reason for admission and type of medication received by the patient), administrator related factors (educational level, work experience, patient-administrator ratio, whether proper administration and documentation was done or not, mediation related factors (type, dose and route of administration), and facility and infrastructure related factors (access of equipment, proper environment, institutional guide, drug information and patient information for medication administration).

### Operational definitions

**Omitted drug error**: when there is failure to administer a prescribed medication

**Unauthorized drug error**: when the prescriber did not authorize the medication administered

**Dose error**: when the medication dose, strength or quantity given is different from that of prescribed

**Patient error**: when a medication of one patient is wrongly given to another patient

**Route error**: when a medication is given on a wrong route of administration

**Time error**: when there is greater than one-hour difference between the ordered time and the time the medication is administered

**Medication administration error (MAE)**: when there is an occurrence of a single or combination of the above listed errors while administering a medication to a patient [17, 18].

### Data management and analysis procedures

After checking the data for its completeness, missing values, and coding of questionnaires, data were entered in to computer and data processing and analysis were done using SPSS version 21 software. Multiple administration errors in a single medication administration were counted as one MAE. Medication administration status was determined for each observed administrations. Data were summarized and described using frequency with percentage for categorical variables and mean with standard deviation for continuous variables. Bivariable and multi-variable logistic regression models with 95% confidence intervals were used to determine the relationship between the independent variables and the dependent variable. Independent variables with *p*-value < 0.3 in the bivariable logistic regression were included into the multi-variable logistic regression model and variables with *P*-value < 0.05 in the final model were considered as independent determinants of MAE. Model fitness was checked by Hosmer Lemeshow test statistics. Data were also presented using tables and graphs.

## Results

### Distribution of medication administration across socio-demographic characteristics

A total of 1251 medication administration was observed from 1251 pediatrics patients with a 97.58% response rate. Observations were made among the patients range between the age of 1 day and 14 years with a mean age of 25.32 months and a standard deviation of 45.36 months. The mean weight of the patients was 8.02 Kilograms with a standard deviation of 8.75 Kilograms. The mean experience level of health professionals in charges of medication administrations was 13.89 months with a standard deviation of 10.84 months. The health professionals in charge of medication administration were taking care of up to 25 patients per day with a mean of 8.73 patients and standard deviation of 7.69 patients per day. About three-fourth (75.6%) of the medications were administered by health professionals who have a Bachelor degree (Table 1).

**Table 1:** Medication administration distribution across socio-demographic characteristic of pediatric inpatients (*n* = 1251)

Characteristic		Number	Percent
Age of patient in completed months	≤ 1	664	53.1
(mean = 2.32 months, SD = 45.36 months)	2–12	199	15.9
	13–60	195	15.6
	> 60	193	15.4
Weight of the patient in complete kilograms	≤ 10	992	79.3
(mean = 8.02 kg and SD = 8.75 kg)	11–20	102	8.2
	> 20	157	12.5
Work experience of health professionals	≤ 12 months	850	67.9
(mean = 13.90 months and SD = 10.84 months)	13–24 months	207	16.6
	> 24 months	194	15.5
Educational level of medication administrator	Student	9	0.7
	Diploma	296	23.7
	Bachelor degree	946	75.6
Patient to medication administrator ratio	≤ 4	295	23.6
	5–10	744	59.5
	> 10	212	16.9

### Distribution of medication administrations across different factors

All administered medications were running with availability of medication card index and without an amount perfuse fixer set. About one third (34.8%) of medication administrations were done in a place where medication preparation room is available. Nearly two-third (64%) of medications was prepared in a place where a computer or medication calculator is available to determine its dose. Six hundred forty seven (51.7%) medications were prepared in a ward, which had no medication administration guide, and 160 (12%) medications were prepared in a ward, which had no documentation system or medication sheet. Almost all (98%) and (93.7%) of the medications observed were administered through intravenous (IV) route and had prepared in the medication room with available standard weight measurement, respectively. Seven hundred eighty-seven (62.9%) medications were observed for patients who had taken two different medications and 769 (61.5%) medications were administered two times (BID) per day (Table 2).

**Table 2:** Medication administration distribution across different factors among pediatric inpatients (*n* = 1251)

Characteristic		Number	Percent
Availability of medication preparation room	Yes	435	34.8
	No	816	65.2
Availability of leveled medication shelf	Yes	856	68.4
	No	395	31.6
Availability of computer or calculator	Yes	801	64
	No	450	36
Availability of medication administration guide	Yes	604	48.3
	No	647	51.7
Availability of standard weight measurement	Yes	1172	93.7
	No	79	6.3
Availability of documentation system	Yes	1091	87.2
	No	160	12.8
Number of medication per a single patient	1	248	19.8
	2	787	62.9
	3 and above	207	16.6
	4 medication	9	0.7
Medication frequency to be administer	When needed (PRN)	6	0.5
	Daily (QD)	226	18.0
	Twice a day (BID)	769	61.5
	Three times a day (TID)	71	5.7
	Four times a day (QID)	163	13
	Every 4 h	16	1.3
Medication administration route	Intravenous (IV)	1226	98.0
	Intramuscular (IM)	8	0.6
	Oral (PO)	17	1.4

### Occurrence and types of medication administration errors

The occurrence of MAE from the total 1251 observed medications administration was 62.7% with a 95% CI (59.6%, 65.0%). The types of MAEs in decreasing their prevalence are administering of wrong dose, administering in the wrong time, medication omission, administering a wrong patient, administering via a wrong route, administering un-prescribed medication and administering a wrong drug, which accounts for 665(85.4%), 429 (55.1%), 18(2.3%), 5(0.6%), 4 (0.5%), 2 (0.3%) and 1(0.1%), respectively (Table 3).

**Table 3:** Occurrence and types of MAE among pediatric inpatients (*n* = 1251)

Characteristic		Number	Percent
Medication administration status	Right medication administration	472	37.3; 95% CI (35.0, 40.0)
	Medication administration error	779	62.7; 95% CI (59.6, 65.0)
Omission of medication ordered	No	1233	98.6
	Yes	18	1.4
Patient drug mismatch	Drug gave to the right patient	1246	99.6
	Drug gave to the wrong patient	5	0.4
Medication type	Right drug	1238	99.9
	Wrong drug	1	0.1
Dose appropriateness	Right does	574	46.3
	Wrong dose	665	53.7
Route of administration	Right route	1235	99.7
	Wrong route	4	0.3
Time appropriateness	Right time	810	65.4
	Wrong time	429	34.6
Prescription status	Prescribed drug	1249	99.8
	Un-prescribed drug	2	0.2

**Fig. 1:**
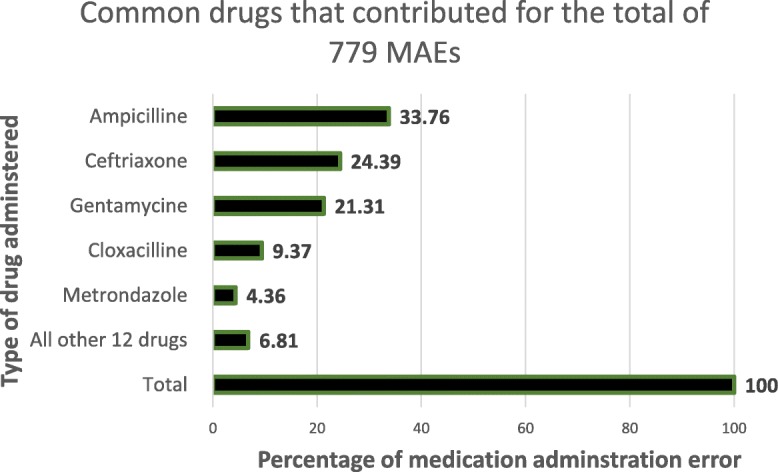
Percentage of types of drugs contributed for the different types of MAEs

The five commonest drugs, which contributed for the MAEs, are ampicillin, ceftriaxone, gentamicin, cloxacilline and metronidazole, with a magnitude of 263(33.76%), 190 (24.39%), 166 (21.31%), 73 (9.37%) and 34 (4.36%), respectively (see Fig. 1).

### Factors associated with medication administration error

From the socio-demographic variables, age of the patients and educational level of health professionals in charge of administering medications were found as significant independent factors associated with MAE.

Medications administered to neonatal patients aged < 1 month, 1–12 months and 13–60 months were about 7.5, 10.8 and 5.7 times more likely to have MAE than patients aged more than 60 months, with AOR(95% CI) of *7.54 (2.20–25.86), 10.84(3.15–37.31)* and *5.69(1.83–17.70), respectively***.** Bachelor degree holder medication administrator health professionals were about 1.5 times higher risk of conducting MAE than diploma holder health professionals with AOR (95% CI) of *1.52(1.07–2.17)*.

Regarding the health facility and drug related variables, availability of medication preparation room, the number of drug prescriptions per patient and availability of medication administration guide were found to be significant independent factors associated with MAE. Medications prepared without the availability of the medication preparation room were about 13.5 times at higher risk of MAE as compared to medications prepared in rooms available for medication preparation with AOR(95% CI) of 13.45 (8.59–21.06). Medications prepared in a place where there is no availability of medication administration guide were about 4 times more likely to have MAE than their counterparts, with AOR(95% CI) of *4.11* (2.89–5.85). Medications which administered among patients who had two prescribed medication types and those who had three or more prescribed medication types were about 2.5 and 1.9 times more likely to have MAE than patients who had single prescribed drug with AOR(95% CI) of 2.46(1.62–3.61) and 1.86(1.14–3.03), respectively. The less experience of health professionals was a factor found to be prevented for MAE. Health professionals in charge of medication administration who have been working in the pediatrics unit for less than 12 months were 63% less likely to commit medication administration error than those experienced above 24 months with AOR (95% CI) of 0.37(0.21–0.65) (Table 4).

**Table 4:** Factors associated with MAE among pediatric inpatients

Variable		MAE		COR (95% CI)	AOR (95% CI)
		Yes	No		
Age of patient in months	> 60	65	128	1	1
	13–60	107	88	2.394 (1.588–3.610)	5.69 (1.83–17.70)^*^
	1–12	155	44	6.937 (4.430–10.864)	10.84 (3.15–37.31)^**^
	< 1	452	212	4.199 (2.988–5.900)	7.54 (2.20–25.86)^**^
Educational level of medication administrators	Diploma	155	155	1	1
	Degree	624	322	1.875 (1.444–2.436)	1.52 (1.07–2.17)^*^
Experience of medication administrators	> 24 months	177	17	1	1
	13–24 months	117	90	0.125 (0.71–0.220)	0.78 (0.41–1.51)
	≤ 12 months	485	365	0.128 (0.76–0.214)	0.37 (0.21–0.65)^**^
Availability of medication preparation room	Yes	146	289	1	1
	No	633	183	6.847 (5.289–8.864)	13.45 (8.59–21.06)^**^
Availability of leveled medication shelf	Yes	502	354	1	1
	No	277	118	1.655 (1.283–2.136)	0.89 (0.11–1.31)
Availability of medication administration guide	Yes	286	318	1	1
	No	493	154	3.559 (2.796–4.531)	4.11 (2.88–5.85)^**^
Number of medication per a single patient	One	126	122	1	1
	Two	523	264	1.918 (1.436–2.562)	2.46 (1.62–3.61)^**^
	Three and above	130	86	1.464 (1.012–2.117)	1.86 (1.14–3.03)^*^

*COR* crude odds ratio, *AOR* adjusted odds ratio, *CI* confidence interval*significant at *p*-value 0.05, **significant at *p*-value 0.01

## Discussion

The study determined the occurrence of medication administration error occurred in all public general hospitals of Tigray, Ethiopia. From the total of 1251 medication administrations, 779 (62.7%, 95% CI: (59.6–65.0%) MAEs was observed. The occurrence of MAE in this study is *consistent* with another study conducted in Nigeria teaching hospital [19], with an incidence or prevalence of 59%. However, the occurrence of MAE in this study is *higher than* that of a study done in France [10, 20], which showed an occurrence rate of *27%*. Similarly, the most common type of medication administration error of both studies was wrong dose. The occurrence of MAE in study is *lower than* the occurrence of MAE found from a study conducted at the Jimma University specialized hospital, Ethiopia, 2010 [12] and another study done in a teaching hospital of India [10, 20] with a rate of 89.9% and 68.5%, respectively. This discrepancy may be because of the difference in educational level, experience level and training of the health professionals and the more developed and equipped facilities and guidelines in the teaching hospital than the general hospitals.

The commonest type of medication responsible for MAE was ampicillin with an error prevalence of 33.8%. This finding is supported by other studies conducted in Jimma University specialized hospital, Ethiopia [12, 21] and in teaching hospital of UK [12, 21] with an occurrence rate of 24.7% and 44%, respectively. This might be related to the frequent administration of ampicillin drug for many diseases than the other drugs. This means that a drug with the highest probability of administration has at the same time high chance of occurrence of MAE.

Adjusting for all other factors, medications administered among pediatric patients less than one-month age, between 1 month and 1 year of age and between 1 year and 5 years of age had higher-risk occurrence of MAE than above 5 years of age. This finding is concordant with the study done in Argentina which showed that infants less than 1 year were 2.61 times more likely to have MAE than above 1 year children [9]. This may point towards the availability of a variety of dosage forms of medications for younger children (infants and neonates) than older children. This might make professionals to be prone to make an error in calculation of dose.

Medications administered by professionals with an educational level of BSc. degree, were 1.52 times more likely to have a risk of MAE than medication administered by diploma holder professionals. This finding is congruent with another study done in a referral hospital of the University of Gondar, Ethiopia, 2016 which shows that medications administered by nurses with the educational level of BSc. degree were 2.51 times more likely to commit MAEs than diploma holders [22]. This important finding of both studies in the country may indicate that the education policy for diploma level is more focused on skill as compared to the degree level, which could more emphasize on theoretical part. The same reason could contribute to the factor in this finding which found that professionals who have longer duration of experience have had committed more MAEs; this could be because freshly employed professionals may have little negligence in preparing and administering medications than senior professionals. A study from Nigerian hospital also concluded that workload was one of the factors that affect the occurrence of MAE [19].

In this study, the higher number of medications given for a single patient had the higher MAE. Patients received two and more than two medications at the same time, are about 2.5 and 2 times more likely to have MAE than patients who receive single medication, respectively. This could be because patients received more than one drug at a time might be prone to confuse professionals in administering the appropriate dosage and time of medication as per the prescription.

Regarding the health facility related factors, lack of availability of medication preparation room and lack of availability of medication administration guide line were found to be significant predictors of MAE. Lack of availability of the medication preparation room/s and lack of availability of medication administration guide/s were about 13 and 4 times more likely to have MAE than their counterparts, respectively. It is obvious that professionals lacked these services would definitely make more MAE.

## Conclusion

The occurrence of medication administration error was found to be high in this study. Age of patients, educational level of medication administrators, availability of medication preparation room and guide and a number of medications given per single patient were statistically significant factors associated with occurrence of medication administration error. Tigray regional health office, medical directors and other responsible bodies of the hospitals should work in providing updated medication administration guidelines, enough space or room for medication preparation, continuous training for health professionals. The health professional should devote their time in updating themselves on how to administer medications to their patients safely and appropriately.

## Additional file

Additional file 1: Questionnaire for medication administration error and associated factors. (DOCX 72 kb)

## Abbreviations

AOR: Adjusted Odds Ratio
CI: Confidence Interval
COR: Crude Odds Ratio
ICU: Intensive Care Unit
MAE: Medication Administration Error
NCC MERP: National Coordinating Council for Medication Error Reporting and Prevention
NIC: Nursing Interventions Classification
WHO: World Health Organization
