# Supplementary material for: Medication administration error and contributing factors among pediatric inpatient in public hospitals of Tigray, northern Ethiopia
Source: BMC Pediatr. 2018 Oct 10;18:321. doi: 10.1186/s12887-018-1294-5 (PMC6180598; doi:10.1186/s12887-018-1294-5)
Supplement: Supplementary file 1 — Questionnaire for medication administration error and associated factors. (DOCX 72 kb) [file 12887_2018_1294_MOESM1_ESM.docx]

## Questionnaire for medication administration error and associated factors

| S.N | Question | | Response | | | Remark | | |
| --- | --- | --- | --- | --- | --- | --- | --- | --- |
| **Part I Socio demographic data** | | | | | | | | |
| 1 | Place of data collection | | Hospital:____________________  Code No:___________________ | | | |  | |
| 2 | Unit of data collection; | | Pediatric ward  Pediatric ICU  Neonatal ICU | | | |  | |
| 3 | Patient information | | 1. age Age Sex Weight | | | |  | |
| 4 | Level of education of the nurse who administer  the medication | | Student  Diploma  Degree  Masters  Other ___________________ | | | |  | |
| 5 | The experience of the nurse in that specific  pediatric unit | | months years | | | |  | |
| 6 | Do you have the workload? | | Yes No  Nurse patient ratio _________ | | | |  | |
| **Part II Medication data** | | | | | | | | |
| 7 | Ordered medication type with prescribed dose,  frequency, route and duration from a cardor  prescription with single does amount to be  administered. | 1. **Medication 1** _________________________ 2. Dose Route Frequency 3. Single dose amount 4. **Medication 2** _________________________   Dose Route Frequency  Single dose amount   1. **Medication 3** _________________________ 2. Dose Route Frequency 3. Single dose amout | | | | |  | |
| **Part III Medication administration equipment and facility data** | | | | | | | | |
| 8 | Does the pediatric unit has a medication  preparation room? | | | | Yes No | |  | |
| 9 | Does the pediatric unit has leveled medicationshelf? | | | | Yes No | |  | |
| 10 | Does the pediatric unit havea computer or  calculator machine to calculate the dose of drug? | | | | Yes No | |  | |
| 11 | Does the pediatric unit havea medication card index? | | | | Yes No | |  | |
| 12 | Does the nurse write the final single does  amount to be administered on the card index? | | | | Yes No | |  | |
| 13 | If the answer for QNo.12 is No; what they write on card index __________________________________  ________________________________________________________________________________ | | | | | | | |
| 14 | Does the pediatric unit have a fluid perfuse  Machine or profuse amount fixer set? | | | Yes No | | |  | |
| 15 | Dose the pediatric unit have guide for  Medication administration? | | | Yes No | | |  | |
| 16 | Does the pediatric unit have standardweight  Measurement? | | | Yes No | | |  | |
| 17 | Does the pediatric unit have after procedure  documentation system? | | | Yes No | | |  | |
| **Part IV Medication administration data** | | | | | | | | |
| 18 | Which types of medication to be administered  froma list of ordered medication type in QNo.7? | | | | 1. ____________________________ | | |  |
| 19 | Does the selected drug to be administer is  similar with the order drug on the card/prescription? | | | | Yes No | | |  |
| 20 | Does the nurse calculate the single dose amount? | | | | Yes No. | | |  |
| 21 | If the answer QNo.20 is yes; Is the calculated dose  similar with the ordered singledose to be  administered in QNo.7? | | | | Yes No  The calculated single dose to be administered | | |  |
| 22 | Does the nurse reconstitute and draw the right  amount of drug does?  Type of serynge used ______________________ | | | | Yes No  The single dose has drawn to be  Administered | | |  |
| 23 | Does the nurse identify the right route and  prepare the patient in appropriate position? | | | | Yes No | | |  |
| 24 | If the answer for QNo. 23 is No; what types of error commit ______________________________ _______ | | | | | | | |
| 25 | Does the nurse give the medication at the right  Time? (with in 30min. befor and after order time) | | | Yes No | | | |  |
| 26 | If the answer for QNo. 25 is No; what types of error commit ________________________________ | | | | | | | |
| 27 | Is there any missed medication does not give to  the patient at a time? | | | Yes No | | | |  |
| 28 | If the answer for QNo. 27 is yes; what type of medication_________________________________ | | | | | | | |
| 29 | Is there any unprescribe drug given to the patient? | | | Yes No | | | |  |
| 30 | If the answer for QNo. 29 is yes; what type of medication _________________________________ | | | | | | | |
| 31 | Is there any drug of this patient misstaklly given to another patient Yes No | | | | | | | |
